# Exosomes Derived From M2 Microglia Cells Attenuates Neuronal Impairment and Mitochondrial Dysfunction in Alzheimer’s Disease Through the PINK1/Parkin Pathway

**DOI:** 10.3389/fncel.2022.874102

**Published:** 2022-04-28

**Authors:** Nan Li, Jun Shu, Xiaoyan Yang, Wenshi Wei, Aijuan Yan

**Affiliations:** Department of Neurology, Cognitive Disorders Center, Huadong Hospital, Fudan University, Shanghai, China

**Keywords:** M2 microglia cells, mitophagy, exosomes, Alzheimer’s disease, Aβ treatment, PINK1/Parkin pathway

## Abstract

The accumulation of abnormal aggregation of amyloid-β plaques is one of the most distinguishing pathologies of Alzheimer’s disease (AD) and is highly toxic to neurons. Exosomes have demonstrated great potential for AD therapy. However, the impact and underlying mechanism of M2 microglia-derived exosomes (M2-EXOs) in AD progression and outcome are seldom explored. Therefore, we employed an Aβ1-42 oligomer (Aβ)-induced AD model in neuronal HT-22 cells and 7-month-old APP/PS1 mice to investigate the effects of M2-EXOs on AD. We revealed that the AD cell model established by Aβ was accompanied by the upregulation of Aβ1-42, neuronal death, alternation of mitochondrial function and autophagy. M2-EXOs can be internalized by HT-22 cells and MAP2-positive neuronal cells in APP/PS1 mice, and exert neuroprotective functions. Specifically, the administration of M2-EXOs in the AD cell model partially increased cell viability, restored the destruction of mitochondrial membrane potential, and reduced the accumulation of reactive oxygen species inside the mitochondria and cells in a dose-dependent manner. Moreover, we demonstrated that PINK1/Parkin mediated mitophagy was enhanced, while incubation with M2-EXOs decreased beclin1, LC3II, PINK1, and Parkin expression levels. Finally, we observed that compared with APP/PS1 mice treated with PBS, the application of M2-EXOs could decrease Aβ plaque deposition and minus Aβ oligomer expression along with improved PINK1/Parkin pathway-mediated autophagy. Overall, our results imply that M2-EXOs play a protective role in the pathogenesis of AD by ameliorating PINK1/Parkin-mediated mitophagy, indicating that it may provide a novel therapeutic strategy to treat AD.

## Introduction

Alzheimer’s disease (AD) is the most common cause of dementia in people over 65 years of age and is one of the most burdening diseases worldwide, with no cure (Gauthier et al., 2021). The distinguishing pathology of AD is the presence of abnormal aggregation of amyloid-β plaques and hyperphosphorylated tau proteins, accompanied by neuronal death and injury to brain tissue, resulting in neurodegeneration and cognitive impairment (Ballard et al., 2011). Nevertheless, the exact pathogenesis of AD remains unclear.

Serving as the resident innate immune cells, microglia can regulate the immune response and mediate crucial functions to dynamically maintain cerebral homeostasis (Butovsky and Weiner, 2018). Microglia exert neurotoxic or neuroprotective functions depending on the polarization phenotype: classic M1 activation and alternative M2 activation (Hu et al., 2014). Mounting evidence suggests that M2 microglia could potently alleviate pathological damage in AD by inhibiting neuroinflammation, promoting tissue repair, and suppressing Aβ deposition and the clustering of neurofibrillary tangles (Edwards et al., 2006; Yao and Zu, 2020).

Exosomes are one specific type of extracellular vesicle, typically with sizes ranging from 30 to 150 nm in diameter, secreted by multiple cells, including microglia (Kalluri and LeBleu, 2020). Increasing evidence indicates that exosomes have a critical impact on mediating the communication of different cells and can exert therapeutic effects in various diseases through specific cargoes (Thakur et al., 2022). Furthermore, previous studies have confirmed that exosomes derived from M2 microglia could attenuate neuronal apoptosis, moderate the formation of the glial scar, thereby relieving ischemic brain damage (Song et al., 2019; Li et al., 2021), but the roles of M2 microglia-derived exosomes (M2-EXOs) in the progression and treatment of AD have seldom been explored.

Mitochondria are essential for cell function and survival, especially in neurons (Lou et al., 2020; Han et al., 2021). Along with the energy crisis, mitochondrial damage is also involved in oxidative stress and cell death, which were considered to participate in the pathogenic mechanism of AD (Vaillant-Beuchot et al., 2021). Mitochondrial homeostasis is governed by mitochondrial biogenesis and mitophagy, the selective elimination of superfluous or functionally impaired mitochondria via autophagy (Pickles et al., 2018). One of the most important mechanisms of mitophagy in neurodegenerative diseases is the PTEN-induced putative kinase1 (PINK1)/Parkin signaling pathway (Quinn et al., 2020).

Based on the aforementioned information, we designed this study to investigate whether M2-EXOs can attenuate mitochondrial damage in AD progression through the mediation of mitophagy via the PINK1/Parkin pathway.

## Materials and Methods

### Establishment of the *in vitro* Alzheimer’s Disease Model

In a humidified incubator at 37°C with 5% CO2, HT-22 cells were grown in Dulbecco’s Modified Eagle Medium (DMEM) (Gibco) with 10% fetal bovine serum (FBS; Gibco) and 1% penicillin/streptomycin. Every 2 days, the complete culture medium was renewed. Before the experiments, cells were seeded in 6-well culture dishes at an appropriate concentration and incubated until they reached 60–70% confluency. The oligomer of amyloid-β protein fragment 1–42 (Aβ) was added to the serum-free culture medium for 48 h at a final concentration of 5 μm/mL.

### M2 Microglial Cell Activation and Identification

BV2 cells were used as a substitute for microglia to obtain M2-EXOs. In a humidified incubator at 37°C with 5% CO2, cells were maintained in DMEM (Gibco) with 10% FBS (Gibco) and 1% penicillin/streptomycin (Gibco). To avoid the influence of exosomes on FBS, cells were briefly cleaned twice using phosphate-buffered saline (PBS) before activation. Subsequently, interleukin 4 (IL-4; PeproTech) was added to the 10% exosome-depleted FBS (SBI) culture medium at a final concentration of 20 ng/mL to activate the M2 phenotype. After 48 h, the medium was then collected for the subsequent isolation of exosomes. Polymerase chain reaction (PCR), Western blot analysis, and immunofluorescence were performed to identify M2 BV2 cells.

### Preparation of the Amyloid-β Protein Fragment 1– 42 Oligomer

The human Aβ1-42 peptide (GL Biochem Ltd.) was dissolved in 1,1,1,3,3,3-hexafluoro-2-propanol (HFIP; Macklin) initially to obtain a 1-mM solution. Subsequently, HFIP was removed using the SpeedVac (Thermo Scientific) to obtain a clear peptide film. The film could be kept at −80°C for 6 months. Before use, the Aβ film was redissolved in anhydrous dimethyl sulfoxide and sonicated for 10 minutes to ensure complete re-suspension, followed by dilution to 100 μm with DMEM. The solution was incubated for at least 24 h and then centrifuged at 13,000 rpm for at least 10 min at 4°C. Finally, Aβ oligomers could be obtained in the supernatant.

### Cell Viability Assay

The viability of HT-22 cells was assessed using the Cell Counting Kit-8 (CCK-8; Dojindo) and Calcein/PI Cell Viability Assay Kit (Beyotime) according to the manufacturer’s protocol. The results of the CCK-8 assay were estimated by the optical density value at 450 nm in each well using a microplate reader (Bio-Rad). Calcein/propidium iodide (PI) staining was evaluated as the number of cells exhibiting green fluorescence divided by the sum of the number of cells displaying green and red fluorescence.

### Isolation, Identification, and Labeling of Exosomes

To remove dead cells, M2 BV2-conditioned medium was centrifuged at 500 *g* for 5 min. Next, to further remove cell debris and particles, the supernatant was centrifuged sequentially at 2000 *g* for 20 min at 10,000 × *g* for 30 min. After ultracentrifugation at 100,000 × *g* for 90 min, exosomes could be initially isolated. PBS was used to wash the isolated exosomes once at 100,000 *g* for 70 min, and the exosomes were resuspended for further characterization. All centrifugations were performed at 4°C.

For M2-EXOs identification, the morphology was captured using transmission electron microscopy (TEM), and the diameter distribution range of particles was examined using nanoparticle tracking analysis (NTA); Western blotting was performed to determine the expression of exosomal surface marker CD63 and tumor susceptibility gene 101 (TSG101).

The red fluorescent membrane dye PKH26 (Sigma) was used to label M2-EXOs according to the manufacturer’s protocol. The labeled exosomes were diluted in PBS for further experiments.

### Detection of Mitochondrial Membrane Potential

The mitochondrial membrane potential (MMP) of HT-22 cells was measured using the mitochondrial membrane potential detection kit (JC-1, Beyotime). Cells with different treatments were stained with the JC-1 staining solution for at least 20 min in the dark at 37°C and then washed using warm wash buffer. Then Leica confocal microscope (Leica SP8) was used to observe the fluorescence of J-aggregates (red) and J-monomers (green) within 30 min.

### Detection of Intracellular and Mitochondrial Reactive Oxygen Species Production

To determine the intracellular accumulation level of reactive oxygen species (ROS), different groups of HT-22 cells were treated with 10-μm dichloro-dihydro-fluorescein diacetate (DCFH-DA; Beyotime) and 200-nm MitoTracker Red (Beyotime) for 20 min in the dark at 37°C. To determine mitochondrial ROS production, 5-μm MitoSOX Red (Invitrogen) and 200-nm MitoTracker Green (Beyotime) were used to stain different groups of HT-22 cells for 20 min in the dark at 37°C. Subsequently, the cells were washed using warm DMEM and observed using a confocal microscope.

### Western Blot Analysis

A radio-immunoprecipitation assay lysis buffer, phenylmethanesulfonyl fluoride, and phosphatase inhibitor cocktail (Beyotime) were utilized to isolate proteins from cells and exosomes. The protein concentrations were evaluated by the BCA Protein Assay Kit (Beyotime) and adjusted. Proteins were separated and transferred onto polyvinylidene difluoride membranes (Millipore). Five percent non-fat powdered milk (Beyotime) was then used to block membranes for 1 h. After that, the membranes were incubated with different primary antibodies against LC3 (1:1000, Abmart), beclin1 (1:2000; Proteintech), Parkin (1:1000; ABclonal), PINK1 (1:1000; Abcam), CD206 (1:1000; Boster), Arginase-1 (Arg-1, 1:1000; Proteintech), CD63 (1:1000; UMIBIO), TSG101 (1:1000; UMIBIO), Aβ oligomer (1:1000, Abcam),Aβ1-42(1:1000,CST) separately overnight at 4°C. Afterward, members were incubated with corresponding secondary antibodies (Beyotime) for 1 hour at room temperature. The enhanced chemiluminescence (Tanon) was used to observe the blots. ImageJ software was used to quantify each band density.

### Immunofluorescence

Cells were resuspended on 35-mm confocal dishes and subjected to different treatments. Then, cells or brain tissue section were washed, fixed, permeabilized, and blocked. Next, they were incubated with different primary antibodies overnight at 4°C (LC3, 1:100; Abmart), Parkin (1:100; ABclonal), PINK1 (1:100; Abcam), CD206 (1:100; R&D Systems), and Arg-1 (1:200; Proteintech), and incubated with corresponding secondary antibodies (1:600; Invitrogen) for 1 h at room temperature. Finally, Cell nuclei were counterstained with the 4′,6-diamidino-2-phenylindole solution. The confocal microscope (TCS-SP8; Leica) was utilized to acquire images.

### Quantitative Real-Time Polymerase Chain Reaction (QRT-PCR)

Total RNA was isolated using the EZ-press RNA Purification Kit (EZB). Then 1000ng of total RNA was reverse transcribed into cDNA by the PrimeScript RT Reagent Kit (TaKaRa) in accordance with the manufacturer’s instructions. SYBR Green Premix Ex Taq (Takara) was utilized to carry out qualitative (q)-PCR with the Stepone Plus Real-Time PCR instrument (Applied Biosystems Life Technology). Fold changes in gene expression levels were estimated using the 2−ΔΔCt method. The primer sequences were listed below: GAPDH, 5′-AGGTCGGTGTGAACGGATTTG-3′ and 5′-TGTAGACCATG TAGTTGAGGTCA-3′; Arg1 5′-GAACACGGCAGTGGCTT TAAC-3′ and 5′-TGCTTAGCTCTGTCTGCTTTGC-3′; and CD206, 5′-TCTTTGCCTTTCCCAGTCTCC-3′ and 5′-TGA CACCCAGCGGAATTTC-3′.

### Animal Experimental Design

7-months-old APP/PS1 double transgenic mice and wild-type mice from the same litter were randomly distributed into three groups: WT group, APP/PS1 group and M2-EXOs treated group, five mice in each group. Specifically, in M2-EXOs group, 100 μg purified M2-EXOs were dissolved in 100 μl PBS and was injected to each APP/PS1 mouse via tail vein once a week. Mice in APP/PS1 group were injected with equal amounts of saline. After 2 months, all mice were anesthetized and transcardially perfused with saline solution, the brains were harvested. Half of each brain was quickly frozen using liquid nitrogen and kept at −80° for protein collection, and the other was fixed, dehydrated and cut into frozen slices at 10 μm for immunostaining.

All animal procedures were approved and carried out in accordance with the recommendations by the Institutional Animal Care and Use Committee of Fudan University.

### Thioflavin S (ThioS) Staining

The prepared slices were soaked in 1% ThioS (Sigma) dissolved in 50% ethanol in the dark for 8 min. After that, the sections were washed twice with 50% ethanol for 10 s each time, and finally with distilled water for 5 min. The results were observed using confocal microscopy and analyzed with image J software.

### Statistical Analyses

Statistical analyses were conducted using Prism GraphPad software (version 9.0). Differences between two groups were evaluated using Student’s *t*-test. Differences among more than two groups were assessed using analysis of variance. All data are presented as means ± standard deviations (SD). *P* value < 0.05 was considered statistically significant.

## Results

### The Establishment of AD Cell Model

**FIGURE 1:**
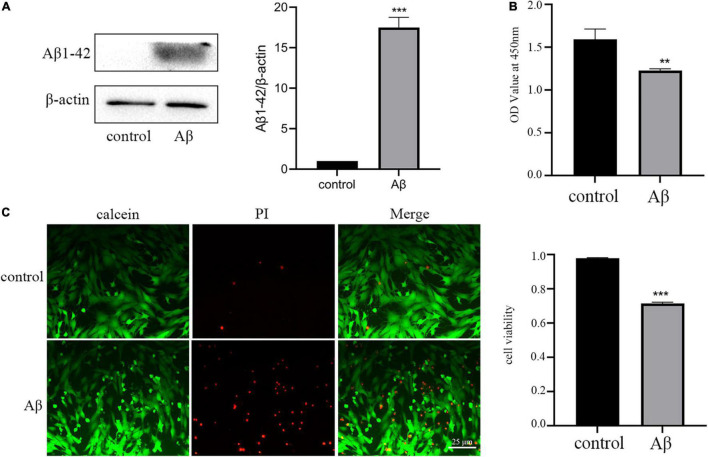
Amyloid-β protein fragment 1–42 (Aβ) treatment upregulation of Aβ1-42 and reduces neuronal viability. **(A)** The expression of AD marker Aβ1-42 detected by Western blot analysis **(B)** Cell viability is determined by the CCK-8 assay. **(C)** Cell viability is measured by calcein/PI staining. All experiments were repeated three times. **P < 0.01 vs. control, ***P < 0.001 vs. control.

Firstly, we established the AD model with the Aβ1–42 oligomer, as previously described (Jian et al., 2019). The results of the Western blot assay demonstrated that incubation of HT-22 cells with 5 μm Aβ for 48 h could markedly upregulate the expression of Aβ1-42 (Figure 1A). Then we used different assays to check the impact of Aβ treatment on the viability of HT-22 cells. The results of the CCK-8 assay illustrated that Aβ significantly reduced the OD values of HT-22 cells (Figure 1B). To visualize the extent of cell damage, we performed a calcein/PI cell viability assay. Calcein-stained living cells displayed green fluorescence, whereas PI-stained dead cells exhibited red fluorescence. Consistent with the results of the CCK-8, we observed that Aβ caused serious cell death (Figure 1C). In short, these data implied that we successfully established AD model with HT-22 cells.

### Aβ Treatment Induced Mitochondrial Impairment and Alternation of Mitophagy

**FIGURE 2:**
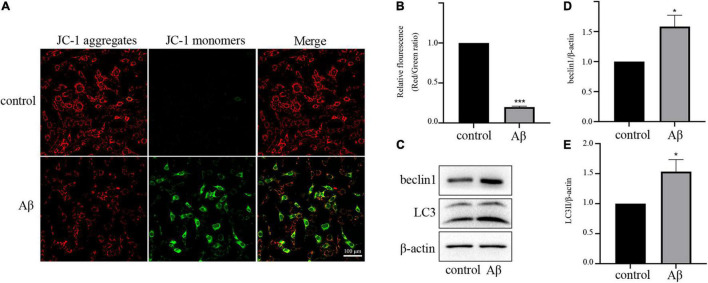
Aβ treatment disrupts the balance of mitochondria and mitophagy **(A)** Representative JC-1 staining images of HT-22 cells. **(B)** Quantitative analysis of the red:green fluorescence intensity. **(C)** Expression levels of mitophagy-related proteins beclin1 and LC3II. **(D)** Quantification of beclin1 expression. **(E)** Quantification of LC3II expression. All experiments were repeated three times. *P < 0.05 vs. control, ***P < 0.001 vs. control.

As the depolarization of the mitochondrial membrane potential can indicate mitochondrial dysfunction, we then determined MMP using a mitochondrial membrane potential assay kit with JC-1. Normally, JC-1 exists as J-aggregates and displays red fluorescence in healthy cells. In mitochondria-damaged cells, JC-1 shifts to a monomer, which displays green fluorescence. As shown in Figures 2A,B, the proportion of cells displaying green fluorescence increased after Aβ treatment, indicating depolarized mitochondria.

Next, we investigated the effect of Aβ on mitophagy, which is strongly linked to mitochondrial quality. For this aim, we used Western blot analysis to determine the levels of autophagy markers LC3II and beclin1. As presented in Figures 2C–E, the expression levels of LC3II and beclin1 proteins were upregulated by Aβ treatment, suggesting the activation of mitophagy. These results suggested that Aβ treatment reduced mitochondrial quantity, and enhanced mitophagy.

### Identification and Characterization of Exosomes Derived From M2 Microglia Cells

**FIGURE 3:**
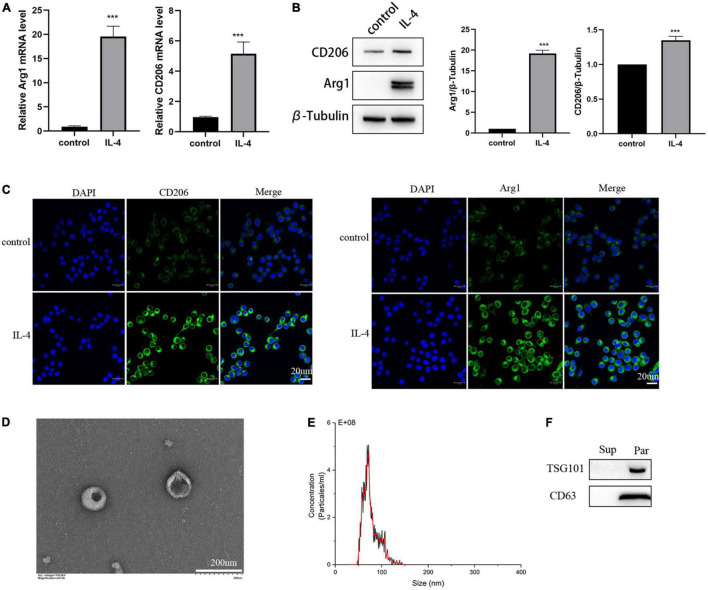
Identification of the isolated polarized M2 microglia and exosomes. **(A)** The expression of M2 microglia markers Arg1 and CD206 detected by RT-PCR. **(B)** The expression of M2 microglia markers Arg1 and CD206 detected by Western blot analysis. **(C)** The expression of M2 microglia markers Arg1 and CD206 detected by immunofluorescence. **(D)** Images of M2-EXOs detected using TEM. **(E)** Measurements of particle sizes range of the isolated exosomes using NTA. **(F)** Detection of exosomal marker proteins CD63 and TSG101 using western blot. All experiments were repeated three times. ***P < 0.001 vs. control.

To investigate whether we successfully isolated exosomes from the M2 microglia-conditioned medium, we first identified the characteristics of M2 microglia cells polarized by IL-4. CD206 and Arg1 are classic M2-polarized microglial markers, and as shown in Figure 3A, results of RT-PCR demonstrated that IL-4 treatment could increase the expression of Arg1 and CD206. The results of Western blot and immunofluorescence analyses also showed that IL-4 stimulation markedly enhanced the expression levels of Arg1 and CD206, implying that IL-4 successfully promoted microglia toward the M2 phenotype (Figures 3B,C).

Then, we isolated exosomes from the M2 microglia-conditioned medium by ultracentrifugation. Under TEM, we observed that the precipitated particles were round and exhibited a typical cup-like structure (Figure 3D). NTA illustrated that the diameter distribution range of the particles was from 30 to150 nm (Figure 3E). Further, Western blot analysis results proved that the specific exosomal biomarkers, including CD63 and TSG101, were substantially expressed in the precipitation (Figure 3F).

In a word, we successfully isolated exosomes from the M2 microglia-conditioned medium by ultracentrifugation.

### M2-EXOs Could Be Phagocytosed by the AD Model and Exert Protective in a Dose-Dependent Way

**FIGURE 4:**
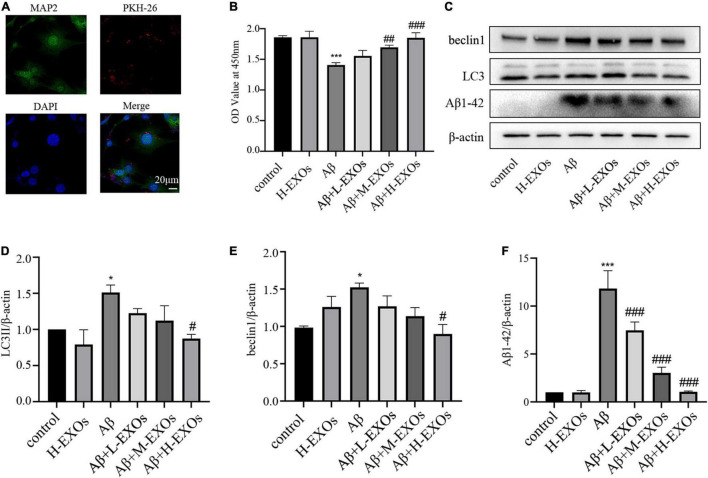
M2-EXOs attenuate the cell impairment caused by Aβ in a dose-dependent way. **(A)** Images of PKH-26-labeled exosomes (red) and neurons (green) after the 24-hour co-culture observed by confocal microscopy. **(B)** The cell viability of HT-22 cells co-cultured with different concentrations of M2-EXOs determined by CCK-8. **(C)** The expressions of LC3II,beclin1 and Aβ1-42 detected by Western blot analysis. **(D)** Quantification of LC3II expression. **(E)** Quantification of beclin1 expression. **(F)** Quantification of Aβ1-42 expression. All experiments were repeated three times. #*P* < 0.05 vs. Aβ, ##*P* < 0.01 vs. Aβ, ###*P* < 0.001 vs. Aβ, *P < 0.05 vs. control, ***P < 0.001 vs. control.

Next, in order to investigate the effects of M2-EXOs on the AD model, we initially need to determine whether M2-EXOs were endocytosed by HT-22 cells. Therefore, PKH26-labeled M2-EXOs were incubated with HT-22 cells and observed after 24 hours. As shown in Figure 4A, PKH26 red fluorescence colocalized with HT-22 cells, suggesting that the M2-EXOs were engulfed by HT-22 cells.

To explore the impact of M2-EXOs on AD *in vitro*, different concentrations of M2-EXOs were added to the AD model. As shown in Figure 4B, M2-EXOs alone had no significant effect on cell viability, while with increasing concentration of M2-EXOs (30-μg/30 × 104 cells in the L-EXOs group, 60-μg/30 × 104 cells in the M-EXOs group, and 90-μg/30 × 104 cells in the H-EXOs group), cell viability of the AD model reversed in a dose-dependent way. Furthermore, M2-EXOs also attenuated the increased expression of LC3II, beclin1 and Aβ1-42 due to Aβ treatment (Figures 4C–F).

To sum up, we initially found that M2-EXOs attenuated Aβ-induced cellular death and mitophagy activation in a dose-dependent manner.

### M2-EXOs Attenuated Mitochondrial Dysfunction of the AD Model

**FIGURE 5:**
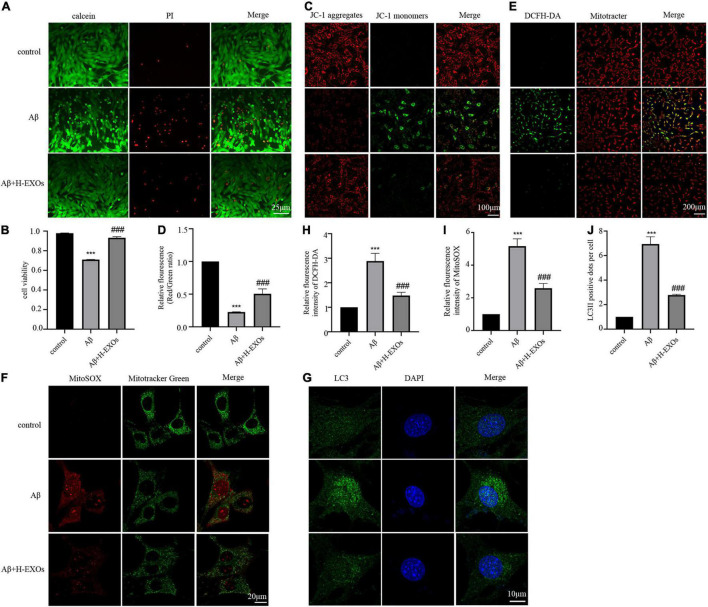
M2-EXOs alleviated cell apoptosis and mitochondrial impairment of the AD cell model. **(A)** Cell viability observed by calcein/PI staining with or without M2-EXO administration. **(B)** Quantitative analysis of Fig A. **(C)** Representative JC-1 staining images with or without M2-EXO administration. **(D)** Quantitative analysis of JC-1 fluorescence intensity. **(E)** Representative DCFH-DA staining images with or without M2-EXO administration. **(F)** Representative MitoSOX staining images with or without M2-EXO administration. **(G)** Representative images of LC3 immunofluorescence with or without M2-EXO administration. **(H–J)** Quantitative analysis of the fluorescence intensity of DCFH-DA, MitoSOX, and LC3. All experiments were repeated three times. ***P < 0.001 vs. control, ###*P* < 0.001 vs. Aβ.

In this section, we explored the impact of M2-EXOs on Aβ-induced mitochondrial damage. As H-EXOs appeared to be the most effective, this concentration was used to perform the following experiments. First, we observed that M2-EXOs reversed the number of dead cells (Figures 5A,B) and loss of MMP (Figures 5C,D) to a normal degree caused by Aβ treatment. We then analyzed the accumulation degree of intracellular and mitochondrial ROS using DCFH-DA and MitoSOX staining, which were quantified as the relative ratio of green to red fluorescence intensity and the relative ratio of red to green fluorescence intensity, respectively. The results showed that the cellular (Figures 5E,H) and mitochondrial ROS (Figures 5F,I) were increased in the AD model, while the application of M2-EXOs attenuated the production of both cellular and mitochondrial ROS. Additionally, the immunofluorescence images revealed that compared with the control group, the number of LC3 punctate dots was markedly decreased in the AD model. However, this effect was alleviated by coculture with M2-EXOs (Figures 5G,J).

In summary, we demonstrated that M2-EXOs could play a protective role in damaged neurons by ameliorating mitochondrial impairment and cell death.

### PINK1/Parkin Pathway Was Involved in the Protective Effects of M2 Microglia-Derived Exosomes

**FIGURE 6:**
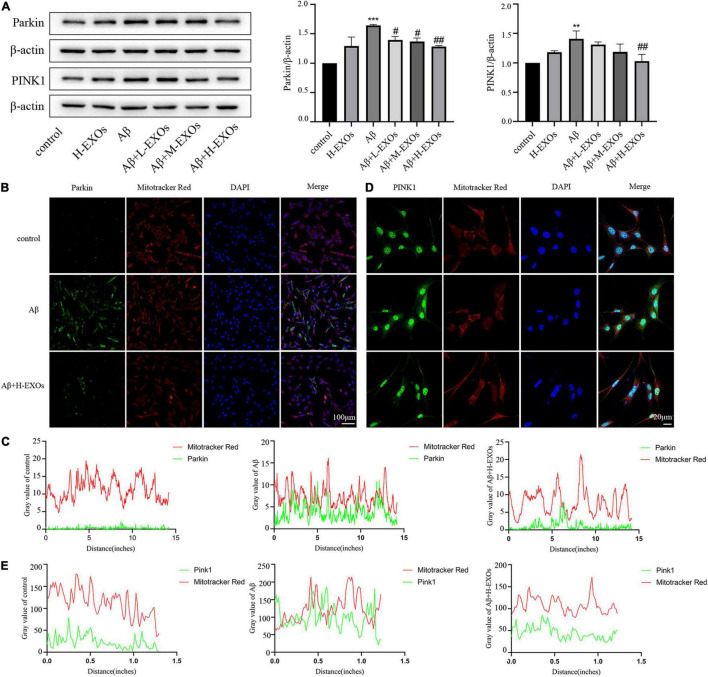
PINK1/Parkin-mediated mitophagy is associated with the protective effects of M2-EXOs. **(A)** Immunoblot images and quantitative analysis of Parkin and PINK1 in the AD model with or without M2-EXO incubation. **(B)** Representative images of Parkin determined by immunofluorescence. **(C)** Colocalization of immunofluorescence signals between Parkin and MitoTracker. **(D)** Representative images of PINK1 determined by immunofluorescence. **(E)** Colocalization of immunofluorescence signals between PINK1 and MitoTracker. All experiments were repeated three times. **P < 0.01 vs. control, ***P < 0.001 vs. control, #*P* < 0.05 vs. Aβ, ##*P* < 0.01 vs. Aβ.

As abundant evidence has revealed that autophagic elimination of injured mitochondria is mainly mediated by the PINK1/Parkin pathway, we examined the impact of the PINK1/Parkin pathway in the AD model. As shown in Figure 6A, we observed that the expressions of PINK1 and Parkin were elevated in the AD model compared to the control cells according to Western blot analysis, while M2-EXOs reduced the expression of PINK1 and Parkin in a dose-dependent manner. Moreover, immunofluorescence results of Parkin and PINK1 and their colocalization analysis with Mitotracker, respectively, further demonstrated that Aβ-induced cell mitophagy was prevented by H-EXOs (Figures 6B–E).

In summary, these findings indicate that the defensive effect of M2-EXOs may be mediated through the PINK1/Parkin pathway.

### M2-EXOs Reduced the Deposition of Aβ in APP/PS1 Mice

**FIGURE 7:**
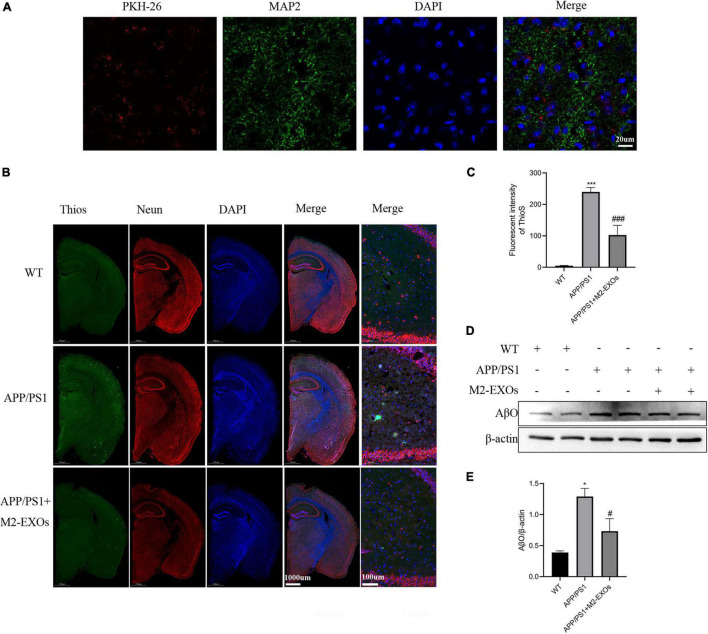
Effect of exosomes on amyloid levels and Aβ-oligomer expression in APP/PS1 mice. **(A)** Images of PKH-26-labeled exosomes (red) and neurons (green) after three days of exosome injection through the tail vein were observed by confocal microscopy. **(B)** Representative images of brains section stained with thioflavin S (ThioS), NeuN, and DAPI. **(C)** Quantitative analysis of Thios fluorescence intensity. **(D)** Immunoblot images of Aβ oligomer in all groups of mice. **(E)** Quantitative analysis of Aβ oligomer. **P < 0.01 vs. WT, ***P < 0.001 vs.WT, #*P* < 0.05 vs. APP/PS1, ###*P* < 0.001 vs. APP/PS1.

Next, we evaluated the role of M2-EXOs in APP/PS1 mice. First, we tested whether exosomes injected into mice through the tail vein could enter the brain. As shown in Figure 7A, 3 days after injection of PKH26-labeled exosomes, we could observe red fluorescence localized with MAP2-positive green fluorescence in the brain sections of mice, indicating that M2-EXOs successfully entered the blood-brain barrier.

Then we assessed the Aβ plaque deposition using Thios staining. Compared with WT mice, Aβ plaques were significantly increased in the brain of PBS treated APP/PS1 control mice, while the administration of M2-EXOs repressed the Aβ plaques deposition levels in the cortex and hippocampus of APP/PS1 mice (Figures 7B,C).

We also performed western blot assay to detect the expression level of Aβ oligomers in the brain of each group of mice. As shown in Figures 7D,E, compared with the WT group, the expression of Aβ oligomers was significantly higher in the hippocampal tissue of APP/PS1 mice treated with PBS, while the APP/PS1 mice treated with M2-EXOs exhibited less expression.

In conclusion, above data illustrated that M2-EXOs could moderate pathological progression in APP/PS1 mice.

### M2-EXOs Improves Mitophagy in APP/PS1 Mice Through Inactivation of PINK1/Parkin Pathway

**FIGURE 8:**
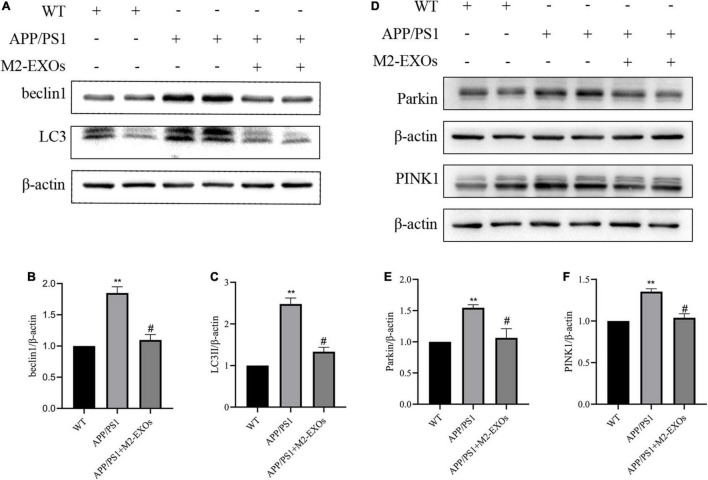
M2-EXOs inhibit autophagy by inactivating PINK1/Parkin signaling in APP/PS1 mice. **(A)** Brain expression of LC3II and beclin1 assessed by western blot analysis. **(B,C)** Quantification of LC3II and beclin1 expression. **(D)** Brain expression of PINK1 and Parkin assessed by western blot analysis. **(E,F)** Quantification of PINK1 and Parkin expression. **P < 0.01 vs. WT, #*P* < 0.05 vs. APP/PS1.

Finally, we determined the alternation of mitophagy in the brain tissue of APP/PS1 mice using Western blot analysis. Consistent with the findings from our *in vitro* experiments, the results confirmed that APP/PS1 mice treated with PBS alone showed the upregulation of LC3II, beclin1, Parkin and PINK1 compared to WT mice. Notably, the upregulation extent of LC3II, beclin1, Parkin and PINK1 in APP/PS1 was significantly reduced by treatment with M2-EXOs (Figures 8A–F). These data indicate that M2-EXOs inhibits the activation of mitophagy pathway in APP/PS1 mice.

## Discussion

In this research, we verified the therapeutic effects of M2-EXOs against Aβ toxicity in HT-22 cells and APP/PS1 mice. We observed that M2-EXOs promoted neuronal survival, restored the destruction of MMP, and reduced ROS production inside the mitochondria and cells. The protective effects of M2-EXOs may be associated with PINK1/Parkin-mediated mitophagy. In addition, we further confirmed the effects of M2-EXOs in APP/PS1 mice.

AD is an insidiously progressive neurodegenerative disease that occurs primarily in the elderly, with progressive cognitive and behavioral impairment as the main clinical manifestation (Wang et al., 2021). Aβ is one of the hallmarks of AD, and extensive studies have shown that Aβ is toxic to neurons and causes cell death (Xiong et al., 2021; Ju and Tam, 2022). Furthermore, multiple lines of evidence have identified Aβ oligomer as crucial cytotoxins associated with AD, as it can damage synaptic function and inhibit long-term potentiation, which is the is the main mechanism responsible for memory decline (Benilova et al., 2012; Tai et al., 2013; Yang et al., 2017). Strategies that can reduce Aβ formation or interrupt their neurotoxicity are badly needed (Tolar et al., 2020). In accordance with previous studies (Litwiniuk et al., 2020; Xiong et al., 2021), our results revealed that Aβ was harmful to cells, which was exemplified by the diminution of CCK8 values and increased PI staining compared with those of the normal group. Cumulative evidence has shown that mitochondrial dysfunction is a critical feature in animal models of AD and in patients with AD (Uddin et al., 2019; Chen et al., 2021). Therefore, we examined the mitochondrial function of cells in the AD model. Consistent with another previous study (Kam et al., 2020), we also found that MMP was greatly reduced in the AD model based on JC-1 staining when compared to the control group. In addition, intracellular ROS was mainly generated in mitochondria, and dysfunctional mitochondria can cause disproportionate ROS generation. Further, previous studies have shown that ROS levels in neuronal cells are upregulated in AD (Butterfield and Halliwell, 2019; Kam et al., 2020). Our results demonstrated that Aβ treatment can stimulate both intracellular and mitochondrial ROS production.

Mitophagy is one type of autophagy that selectively eliminates dysfunctional mitochondria and is critical for mitochondrial quality control. Increasing evidence has revealed that abnormal mitophagy occurs in the brains of patients with AD. Both *in vitro* and *in vivo* studies have confirmed that Aβ can disrupt the dynamics of mitophagy (Kam et al., 2020; Chen et al., 2021). Consistent with other studies (Kam et al., 2020; Sun et al., 2020), we found that the expression of the mitophagy markers LC3II and beclin1 increased in the AD model, according to Western blot analysis.

Recently, exosomes have emerged as a novel and promising candidate approach for AD therapy owing to their unique advantages (Zhang N. et al., 2021; Zhang T. et al., 2021). Specifically, exosomes are stable in physiological fluids and can cross the blood-brain barrier, secrete various proteins and RNAs, and mediate the mutual communication between cells. Furthermore, accumulating evidence has shown that microglia-derived exosomes can regulate glial scar formation, inhibit neuronal inflammation, and promote neurite outgrowth after stroke (Huang et al., 2018; Li et al., 2021). However, the understanding of the beneficial effects of M2-EXOs on AD is in the early stages. Here, we successfully isolated exosomes from the conditioned medium of M2 microglia and observed that M2-EXOs administration increased cell viability, restored the disruption of MMP, and decreased the expressions of LC3II, beclin1 and Aβ1-42.

In neural cells, PINK1/Parkin mediated signaling is the most dominant signaling pathway of mitophagy. Besides, Kam et al. found that the expression level of Parkin was enhanced in the hippocampi of 3 × Tg mice with AD (Kam et al., 2020). Consistent with their findings, we also observed overexpression of PINK1 and Parkin in the AD cell model and 7-month-old APP/PS1 mice, as determined by Western blot and immunofluorescence analyses, while the administration of M2-EXOs decreased their expression in a dose-dependent manner. Thus, we speculated that M2-EXOs may exert their protective role through the Pink1/Parkin pathway.

In conclusion, our study’s results indicate that M2-EXOs can restore cell apoptosis, alleviate mitochondrial function, and reduce ROS production by improving PINK1-Parkin-mediated mitophagy, and these data may offer new insights into the therapeutic effect of M2-EXOs on AD.

Despite great advances in our understanding of how M2-EXOs could restore AD model injury, the detailed mechanism of the events taking place remains limited. As exosomes exert their function by transferring proteins, messenger RNAs, and micro RNAs, in this respect, Huang et al. demonstrated that the expression level of miR-124-3p elevated in microglia-derived exosomes after traumatic brain injury. Besides, exosomal miR-124-3p could suppress inflammation in neuron cells and promote neuronal growth (Huang et al., 2018). In addition, the protective effects of M2-EXOs in cerebrovascular disease reported by Song and Li et al. were also carried out through miR-124 (Song et al., 2019; Li et al., 2021). Furthermore, Du et al. found that miR-124 could alter autophagy levels and alleviates AD pathological progression in APP/PS1 mice (Du et al., 2017). Therefore, we speculate that the protective role of M2-EXOs in AD may be mediated through miR-124. miRNA microarray analyses are warranted to identify the altered miRNA in M2-EXOs and detailed studies are needed to confirm our speculations about the specific mechanisms behind the neuroprotective effect of M2-EXOs.

## Data Availability Statement

The original contributions presented in the study are included in the article/supplementary material, further inquiries can be directed to the corresponding authors.

## Ethics Statement

The animal study was reviewed and approved by Institutional Animal Care and Use Committee of Fudan University.

## Author Contributions

WW and NL were responsible for study design. NL, JS, XY, and AY performed the experiments. AY provided technical support. NL and JS interpreted the results, performed data analysis, prepared the figures and tables, and wrote the manuscript. All authors have read and approved the manuscript.

## Conflict of Interest

The authors declare that the research was conducted in the absence of any commercial or financial relationships that could be construed as a potential conflict of interest.

## Publisher’s Note

All claims expressed in this article are solely those of the authors and do not necessarily represent those of their affiliated organizations, or those of the publisher, the editors and the reviewers. Any product that may be evaluated in this article, or claim that may be made by its manufacturer, is not guaranteed or endorsed by the publisher.
